# From Tea to Topical Agent: Machine Learning and Bioinformatics Reveal KU DING Tea’s Anti-UV Ingredients and Mechanisms

**DOI:** 10.3390/ph19010028

**Published:** 2025-12-22

**Authors:** Jing Huang, Mingzhi Zhang, Xiangling Qin, Qi Yang, Jinling Xie, Xiaotao Hou, Erwei Hao, Jiagang Deng, Zhengcai Du

**Affiliations:** 1 Institute of Chinese Medicine, Zhuang and Yao Medicine, Guangxi University of Chinese Medicine, Nanning 530200, China; huangjing2023@stu.gxtcmu.edu.cn (J.H.); qinxiangling2023@stu.gxtcmu.edu.cn (X.Q.); 13257716536@163.com (J.X.); xthou@126.com (X.H.); Haoew@gxtcmu.edu.cn (E.H.); 2 Key Laboratory of Pharmacodynamic Research of Chinese Medicine in Guangxi, Guangxi University of Chinese Medicine, Nanning 530200, China; yangqi2023@stu.gxtcmu.edu.cn; 3 University Engineering Research Center of Reutilization of Traditional Chinese Medicine Resources, Guangxi University of Chinese Medicine, Nanning 530200, China; 4 Key Laboratory of Theory and Transformation of Chinese Medicine for Dampness and Wetness Diseases in Guangxi, Guangxi University of Chinese Medicine, Nanning 530200, China; 5 International Zhuang Medical Hospital, Guangxi University of Chinese Medicine, Nanning 530200, China; zhangmingzhi2023@stu.gxtcmu.edu.cn; 6 Faculty of Pharmacy, Guangxi University of Chinese Medicine, Nanning 530200, China

**Keywords:** KU DING tea, UV damage, machine learning, network pharmacology, AGE-RAGE signaling pathway

## Abstract

**Objectives**: KU DING tea is a traditional Chinese herbal tea traditionally used topically for inflammation. This study aimed to investigate its potential anti-UV effects. **Methods**: The chemical components of KU DING tea were identified using UHPLC-Q-TOF-MS. Permeability prediction was performed to assess transdermal potential. A machine learning was applied to predict anti-UV activity, and network pharmacology analysis was used to explore the potential mechanism of action. **Result**: A total of 76 chemical components were identified, with 21 predicted to have good transdermal potential. A machine learning Random Forest (RF) model (accuracy 0.84, F1 0.84, AUC 0.93) predicted components like salicylic acid and methyl salicylate likely possess significant anti-UV activity. Network pharmacology indicated the mechanism may involve targets MAPK14 and NFKB1, influencing the AGE-RAGE signaling pathway. **Conclusions**: KU DING tea is a promising natural and safe anti-UV agent, deserving further experimental validation.

## 1. Introduction

Ultraviolet (UV) radiation profoundly impacts human health by inducing DNA damage. UVB (280–315 nm) directly causes DNA crosslinking lesions such as cyclobutane pyrimidine dimers (CPDs) and 6-4 photoproducts (6-4PPs), while UVA (315–400 nm) indirectly generates reactive oxygen species (ROS), leading to oxidative stress and single-strand breaks [1]. These damages not only hinder DNA replication and transcription but are also closely associated with skin cancers (e.g., basal cell carcinoma, squamous cell carcinoma), photoaging, ocular disorders (e.g., cataracts, pterygium), and immunosuppression [2]. Current protective strategies primarily rely on sunscreens and phototherapy drugs, which have significant limitations. Chemical UV filters like oxybenzone may induce phototoxic reactions, triggering cell death or chronic skin inflammation [3]. Certain medications (e.g., hydrochlorothiazide) can even increase skin cancer risk by disrupting the p53-MDM2 signaling axis [4]. Therefore, developing natural, multi-target, and high-safety anti-UV agents has become an urgent priority.

KU DING tea (Ilex kudingcha C. J. Tseng), a widely consumed herbal tea in China, exhibits excellent safety profiles [5]. Beyond its nutritional value, it possesses various health benefits and has been traditionally used topically for its anti-inflammatory effects [6], suggesting its potential as an adjunctive therapy for UV-induced damage. Modern research indicates that KU DING tea is rich in flavonoids (e.g., quercetin, kaempferol) [7], triterpenes (e.g., ursolic acid) [8], and polyphenols [9], demonstrating broad-spectrum UV absorption capacity. Its extracts show over fivefold higher absorbance in the 290–400 nm range compared to synthetic sunscreens [10]. A study by Henan University revealed that KU DING tea flavonoids alleviate UVB-induced keratinocyte damage by suppressing inflammatory cytokines such as IL-6 and TNF-α [11]. However, existing research has focused primarily on flavonoids rather than the full spectrum of its constituents, and the underlying anti-UV mechanisms remain unclear.

Breakthroughs in machine learning have introduced new paradigms for analyzing complex biological systems. By leveraging structure–activity relationships, machine learning predictive models can efficiently screen potential lead compounds in early-stage drug development [12]. Meanwhile, network pharmacology elucidates the multi-component synergistic mechanisms of natural medicines by constructing “disease-gene-compound” interaction networks, playing a pivotal role in traditional medicine research [13]. Despite their individual strengths, the integration of these approaches remains nascent. While machine learning models uncover hidden associations, they lack dynamic validation of biological pathways, whereas network pharmacology relies on static condition-matching analyses without in-depth molecular structural exploration.

This study proposes, for the first time, a four-dimensional framework combining UHPLC-Q-TOF-MS-based component identification, machine learning-driven target prediction, network pharmacology pathway integration, and molecular docking calculations analysis. First, UHPLC-Q-TOF-MS was employed to comprehensively characterize KU DING tea’s chemical profile, followed by ADME-based screening for transdermally permeable compounds. Next, a machine learning model was built to predict anti-UV activity, while network pharmacology unraveled potential molecular mechanisms. Finally, molecular docking validated binding interactions. This systematic strategy provides novel insights into KU DING tea’s anti-UV potential, paving the way for further research.

## 2. Results

### 2.1. Identification of Chemical Components in KU DING Tea

**Figure 1:**
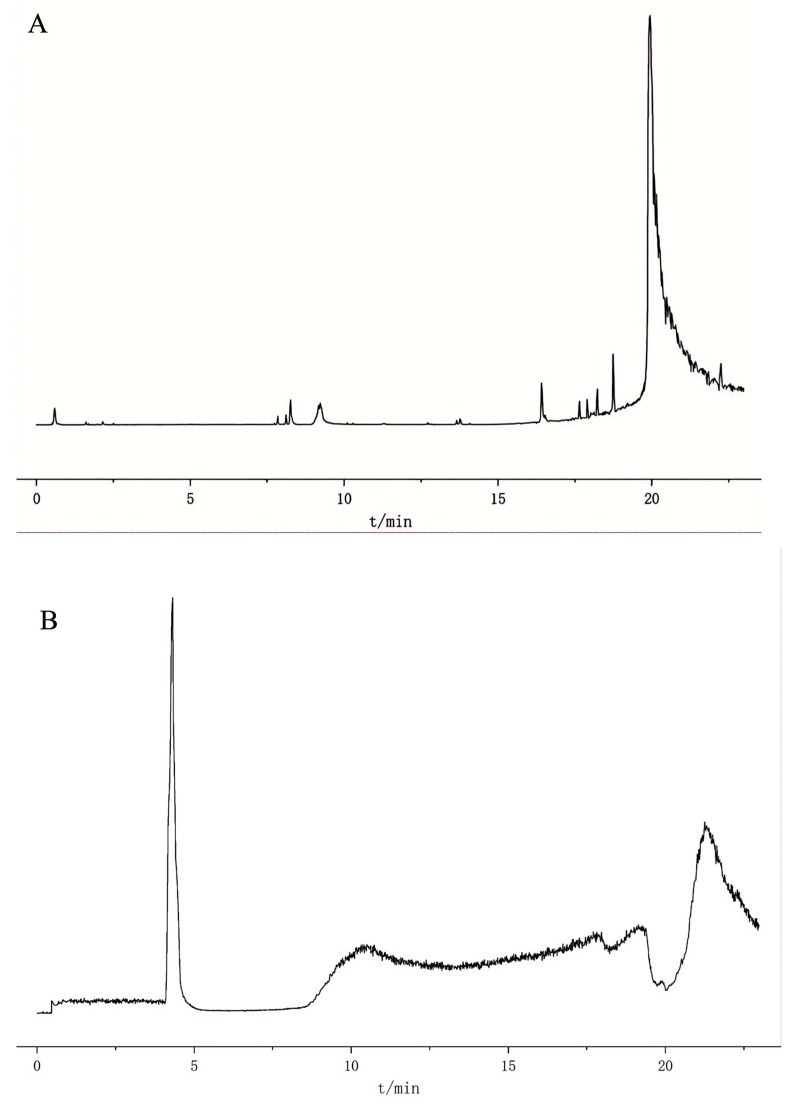
Base Peak Intensity Chromatogram (BPI) of the KU DING tea samples analysis by UHPLC-Q-TOF-MS, corresponding to positive ion mode (**A**) and negative ion mode (**B**).

**Figure 2:**
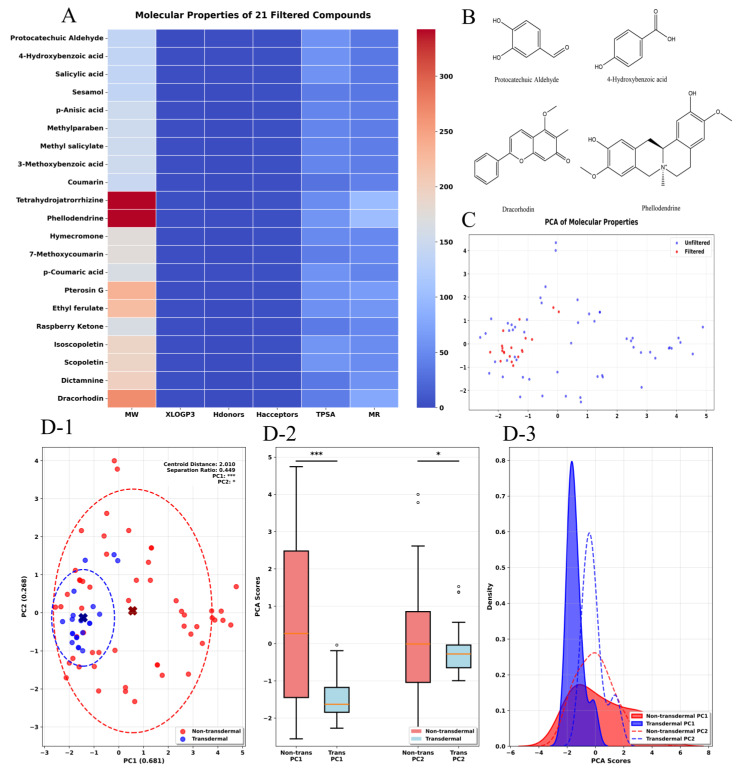
Presents three complementary visualizations. (**A**) displays a heatmap of the 21 selected compounds and their corresponding transdermal properties, where color intensity reflects property values (red indicating higher values, blue indicating lower values); (**B**) shows the molecular structures of four representative compounds with particularly promising transdermal potential. (**C**) shows the transdermal-permeable compounds (red) and others (blue); (**D-1**) depicting separation of group centroids by MANOVA. (**D-2**) group differences on PC1 and PC2 by Bonferroni-corrected *t*-tests; (**D-3**) distribution patterns with partial overlap by kernel density estimation.

**Figure 3:**
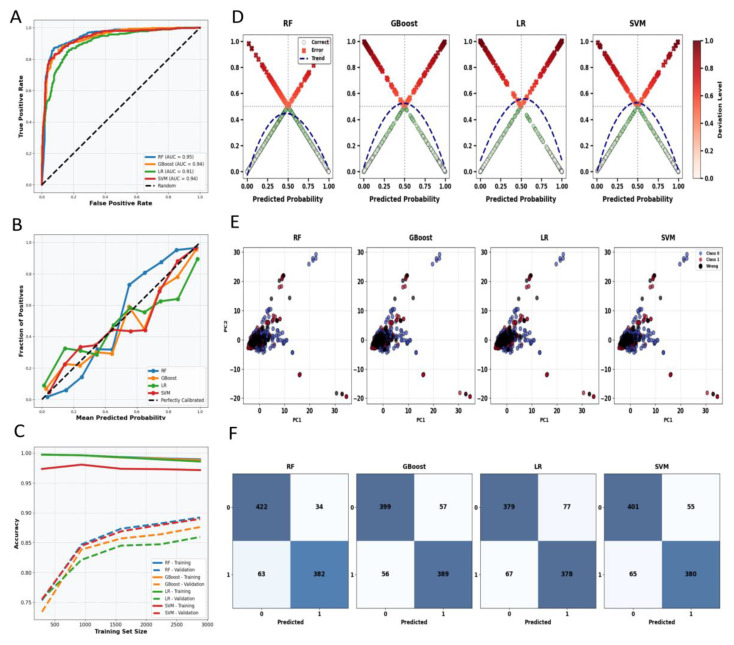
Shows performance evaluation of four machine learning algorithms (RF, GB, LR, and SVM). (**A**) ROC curves; (**B**) calibration curves; (**C**) learning curves; (**D**) prediction bias analysis; (**E**) two-dimensional scatter distribution of compounds’ 1024-bit Morgan fingerprints, with different colors representing distinct annotation classifications; (**F**) confusion matrix.

KU DING tea solution was analyzed in both positive and negative ionization modes, and the resulting Base Peak Intensity Chromatogram (BPI) is presented in Figure 1. We employed UHPLC-Q-TOF-MS to acquire accurate molecular mass data on the chemical constituents of KU DING tea. In total, 75 compounds were identified, with 63 and 12 compounds detected in the positive and negative ion modes, as summarized in Table 1. Among these, 21 compounds including Protocatechuic Aldehyde, 4-Hydroxybenzoic acid, and Salicylic acid, satisfied the transdermal permeability screening criteria outlined in Section 4.1.6. Furthermore, Figure 2A,B illustrates the relatively homogeneous distribution of the 21 potential transdermal compounds across the six transdermal permeability-related molecular descriptors. These compounds also exhibited a clustered distribution in the PCA-reduced dimensionality space derived from principal component analysis. Figure 2(D-1) demonstrates a significant difference in the centroid positions between the “transdermal” and “non-transdermal” compound groups within the PCA space, as revealed by multivariate analysis of MANOVA (*p* < 0.05). Figure 2(D-2), Bonferroni-corrected *t*-tests revealed significant group differences on both principal components PC1 and PC2 (*p* < 0.05). Figure 2(D-3) visually demonstrates the separation trend and partial overlap of the two groups within the chemical space, collectively validating the significance of their distribution differences from both statistical and visual perspectives. Accordingly, these 21 compounds were thus identified for subsequent in-depth analysis.

### 2.2. Performance of Machine Learning Prediction Models

The predictive performance of four machine learning algorithms (RF, GB, LR, and SVM) was comprehensively evaluated using multiple metrics. Figure 3 shows the ROC curve (Figure 3A) and calibration curves (Figure 3B), their generalization capability and regression performance, respectively; the learning curve (Figure 3C) demonstrated the trend in training and testing set accuracy as the number of training samples increases; Figure 3D quantifies and visualizes the prediction bias of each algorithm. Additionally, a two-dimensional distribution plot of compounds based on 1024-bit Morgan fingerprints (E) intuitively displayed chemical space features, while the confusion matrix (F) detailed classification results for positive and negative labels.

The ROC curve (Figure 3A) shows that the curves for Random Forest and Decision Tree are closer to the upper-left corner, indicating superior generalization capabilities compared to Logistic Regression and Support Vector Machine. This aligns with the learning curve results (Figure 3C): Random Forest and Decision Tree exhibit a smaller gap between training and testing accuracy, reflecting stronger resistance to overfitting. The calibration curve (Figure 3B) further indicates that GB and LR exhibit better fit, meaning their predicted probabilities are closer to the true correct probabilities. The prediction bias analysis (Figure 3D) and confusion matrix (Figure 3F) collectively demonstrate that GB achieves a superior balance, showing minimal error bias and strong discrimination capability in both positive and negative categories. The unique clustering patterns revealed in the fingerprint visualization (Figure 3E) provide a structured interpretation of algorithmic performance, demonstrating the model’s ability to distinguish defined chemical spaces. As shown in Table 2, all four machine learning models exhibit excellent performance in predicting UV resistance, with accuracy exceeding 0.80 and AUC values above 0.85, establishing a robust baseline for predictive capability. Among these, the Random Forest model stood out, demonstrating balanced superiority across all evaluation metrics: accuracy (0.84), F1 score (0.84), AUC (0.93), sensitivity (0.85), specificity (0.83), precision (0.86), and MCC (0.68).

### 2.3. Prediction Results

**Figure 4:**
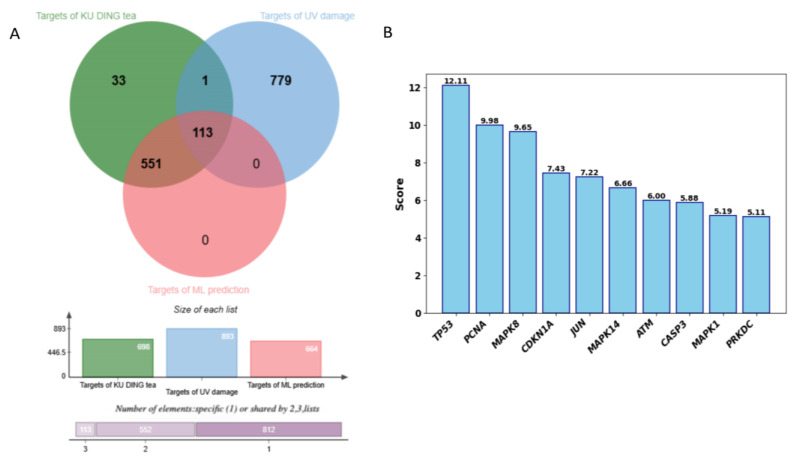
(**A**) presents a Venn with bar charts depicting with target distribution and overlaps among KU DING tea, UV-induced damage, and transdermal components. (**B**) displays the relevance scores of targets common to all three groups, displayed in descending order.

Among the 21 potentially transdermal compounds, 13 showed mean prediction probabilities > 0.5 across all models, with 5 compounds exceeding 0.95: Salicylic acid (1.00), Methyl salicylate (1.00), Raspberry Ketone (0.96), *p*-Coumaric acid (0.95), and 4-Hydroxybenzoic acid (0.95) (Table 3). Subsequent analysis of these 13 compounds through BATMAN-TCM identified 664 targets, with 133 targets emerging from the three-way intersection analysis. Notably, TP53 was the highest-scoring common target (Figure 4).

### 2.4. KEGG Signaling Pathway Analysis

**Figure 5:**
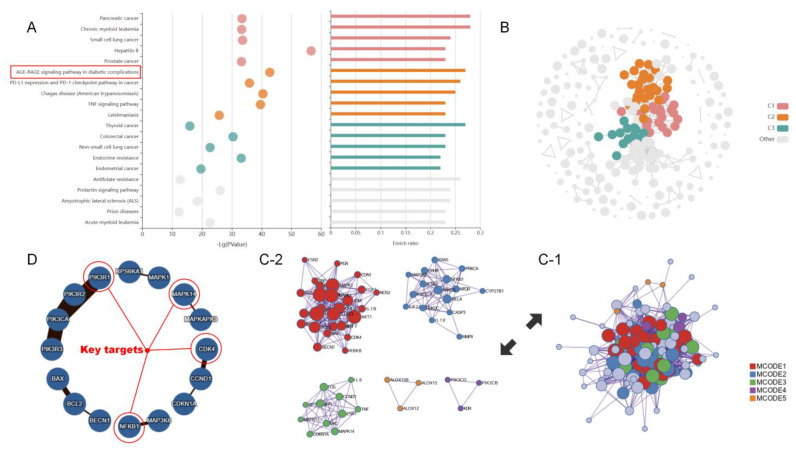
(**A**) displays the top 20 enriched KEGG pathways by −log10(*p*-value). (**B**) shows four-category clustering analysis of these pathways, where different colors represent distinct clusters. (**C**,**D**) show protein–protein interaction (PPI) networks and key target within the AGE-RAGE signaling pathway. (**C-1**) illustrates the overall PPI network clustering of KEGG-related genes, while (**C-2**) breaks down the four individual clusters.

Analysis of the 133 targets revealed several significant pathways (Figure 5), with the AGE-RAGE signaling pathway emerging as particularly noteworthy (ranked 6th in enrichment analysis, (Figure 5A). The protein–protein interaction network (Figure 5C) and pathway-specific target mapping (Figure 5D) identified four key targets in the AGE-RAGE pathway that formed six compound–target pairs for molecular docking: 4-Methoxybenzoic acid-MAPK14, P-Coumaric Acid-NFKB1, Salicylic Acid-NFKB1, Methyl 4-Hydroxybenzoate-PIK3R1, Methyl Salicylate-PIK3R1, and Salicylic Acid-CDK4.

### 2.5. Molecular Interaction Analysis

**Figure 6:**
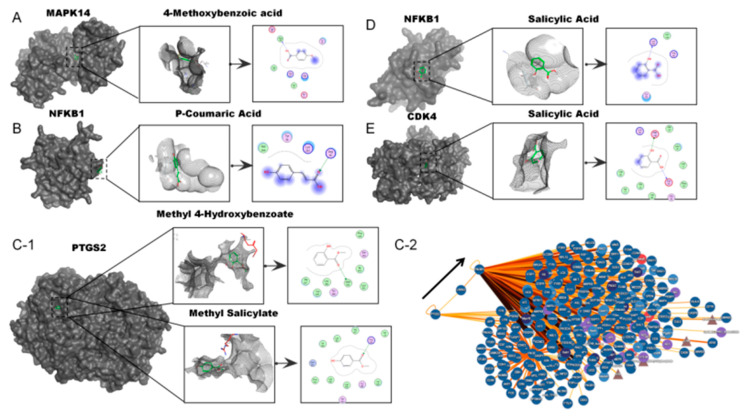
(**A**–**E**) sequentially depict the ligand’s binding position within the protein, the visualization of the binding pocket, and detailed interaction analysis. (**C-2**) reveals the regulatory relationship between PTGS2 and PIK3R1, as derived from PPI network analysis.

Molecular docking results (Figure 6 and Table 4) demonstrated binding for all compound–target pairs, with docking scores below −3.0 (lower scores indicating better binding). Key interactions included 4-Methoxybenzoic acid forming hydrogen bonds with PHE169 in MAPK14; P-Coumaric Acid and Salicylic Acid interacting with ARG56 and LYS243 in NFKB1; Methyl 4-Hydroxybenzoate and Methyl Salicylate potentially acetylating Ser530 in PIK3R1 similar to aspirin’s mechanism; and Salicylic Acid forming stable interactions with ASP158 and GLU94 in CDK4. These findings support our hypothesis about PTGS2’s regulatory role on PIK3R1.

## 3. Discussion

In current pharmacological research on complex natural medicines, network pharmacology is a widely used analytical approach. However, most existing studies retrieve the chemical components of target natural medicines from public databases [18], which inevitably leads to discrepancies between different databases and may misguide subsequent experimental designs [19]. For example, the TCMSP database lists 94 chemical components for KU DING tea (as of 28 May 2025) [20]; however, none of these constituents overlap with the 75 compounds identified in this study. Relying solely on database records could introduce significant prediction errors, underscoring the necessity of proper compound identification before conducting network pharmacology analysis.

When screening active ingredients in natural medicines, most studies still focus on oral bioavailability (OB) [21,22]. However, for anti-UV effects that primarily act on the skin surface, OB may not represent the most pertinent parameter. Acknowledging this constraint, we incorporated transdermal potential prediction after compound identification. Recently, emerging studies have started to call for increased emphasis on transdermal properties in skin-relevant pharmacological models [23]. Consequently, following the identification of chemical components of KU DING tea, transdermal potential evaluations were carried out to supply more accurate and dependable candidate molecules for subsequent investigation of its topical efficacy mechanisms.

Compared with the extant literature, previous research on KU DING tea has primarily focused on phenotypic effects, such as the free radical scavenging activity of KU DING tea polyphenols [24], without providing a systematic delineation of its multi-pathway regulatory characteristics. In contrast, the present study adopts an integrative approach that combines compound identification, prediction of transdermal permeation potential, and network pharmacology analysis to establish a comprehensive multi-level framework—“component–target–pathway”—for the photoprotective effects of KU DING tea. This framework addresses existing search gaps, which have predominantly centered on single components or isolated effects, by elucidating the coordinated regulatory mechanisms underlying its UV-protective actions.

To enhance the reliability of our findings, we incorporated machine learning predictions based on the “structure-activity relationship” theory. By training the AI model with structurally known anti-UV drugs, the predicted compounds were expected to share similar molecular properties or structural features, implying analogous biological effects [25]. The intersection of “component-target,” “disease-target,” and “structure-target” further improved the scientific rigor of target prediction.

Bioinformatics analysis revealed that the AGE-RAGE signaling pathway may be central to KU DING tea’s anti-UV effects. Recent studies have confirmed this pathway’s role in three key aspects: oxidative stress and inflammatory responses [26], AGEs formation and RAGE upregulation [27], and melanogenesis and pigmentation [28]. Molecular docking analyses suggested that multiple active constituents in KU DING tea could exert anti-UV-damage effects via synergistic modulation of pivotal nodes within the AGE-RAGE signaling network. Specifically, 4-methoxybenzoic acid potentially targets MAPK14, thereby suppressing UV-induced phosphorylation and activation of p38 MAPK, and subsequently inhibiting downstream inflammatory signaling transduction [14]. The co-action of p-coumaric acid and salicylic acid on NFKB1 may impede the nuclear translocation of the NF-κB transcription factor, leading to attenuation of the inflammatory cascade elicited by ultraviolet irradiation [15,16]. Concurrently, salicylic acid-mediated inhibition of CDK4 could mitigate UV-induced cell cycle abnormalities, thereby reducing aberrant proliferation of damaged cells [17]. Through these synergistic multi-target effects, these constituents modulate the MAPK-NF-κB signaling axis within the AGE-RAGE pathway and influence downstream inflammatory mediator expression. This molecular-level, multi-pathway inhibition of UV damage offers a novel mechanistic explanation for the anti-photoaging properties attributed to KU DING tea.

In addition, KU DING tea, as a natural product with relatively favorable safety, contains constituents at lower concentrations, suggesting a potentially lower risk of adverse effects compared with synthetic drugs and indicating promising prospects for application.

This study has several limitations. Although multivariate statistical analysis revealed global distributional differences in chemical space between the “transdermal” and “non-transdermal” groups, the moderate separation between their centroids indicates limited precision in the physiochemical descriptor-based model for predicting transdermal potential. Furthermore, experimental validation of transdermal permeability, anti-UV efficacy, and related mechanisms has not been conducted. Future studies should integrate in vitro skin permeation assays and transdermal pharmacokinetic analyses to empirically verify the predicted results. However, it should be noted that changes in transdermally permeable compounds could alter the predicted targets and pathways.

## 4. Materials and Methods

### 4.1. Identification of Chemical Components in KU DING Tea Rapid Identification of Chemical Components in KU DING Tea Using UHPLC-Q-TOF-MS

#### 4.1.1. Instruments and Reagents

The analysis was performed using a SCIEX X500R QTOF high-resolution mass spectrometer (AB SCIEX, Framingham, MA, USA). Ultra-pure water and acetonitrile (both MS-grade) were obtained from Sigma-Aldrich (St. Louis, MO, USA). Data processing was conducted using SCIEX OS 2.0 software (SCIEX, Framingham, MA, USA).

#### 4.1.2. Preparation of Test and Reference Solutions

Test sample: Kudingcha shreds, add 15 times the amount of water for the first time, soak for half an hour, heat to boiling and extract for 1 h, add 13 times the amount of water for the second time, extract for 1 h, combine the two decoctions, and concentrate to a certain concentration. Take the supernatant, centrifuge at 3500 rpm for 10 min, take the supernatant again, and set aside.

#### 4.1.3. Chromatographic Conditions

Chromatographic conditions: Chromatographic separation was achieved using a Phenomenex column (100 × 2.1 mm, 1.7 µm, Phenomenex, Torrance, USA) maintained at 40 °C. The mobile phase consisted of 0.1% formic acid in water (A) and acetonitrile (B), with a flow rate of 0.4 mL/min. The elution gradient was programmed as follows: 5% to 95% B over 20 min, followed by holding at 95% B for 3 min. The injection volume was 3 µL, and the sample tray temperature was set at 4 °C.

#### 4.1.4. Mass Spectrometric Conditions

Mass spectrometric conditions: Analysis was performed using a dual-spray TurboV ion source (SCIEX, Framingham, MA, USA)in both positive and negative ion modes. The mass scan range was set at *m*/*z* 100–1500. The ion source parameters were as follows: positive ion voltage at 5500 V, negative ion voltage at −4500 V, ion source temperature at 600 °C, nebulizer gas pressure at 55 psi, auxiliary gas pressure at 55 psi, and curtain gas pressure at 35 psi. The collision energy for precursor ions was set at −10 V, declustering potential at −80 V, and collision energy for product ions at −35 V.

#### 4.1.5. Data Processing

The acquired mass spectrometry data were imported into the “Analytics” module of SCIEX OS 2.0 software. Identification was performed using the TCM MS/MS Library 2.1 containing approximately 2000 compounds in non-target mode. The matching criteria included a mass error of less than 15 ppm (weight score 20), fragment mass error of less than 15 ppm (weight score 20), library hit score above 70 (weight score 40), and formula finder score above 50 (weight score 20) using Smart Confirmation matching mode. Compounds with a total weight score exceeding 10 were considered valid identifications.

#### 4.1.6. Prediction of Transdermal Absorption Potential

All identified chemical components were analyzed for their absorption potential using the ADME function on Swiss Target Prediction. The evaluation considered molecular weight (less than 500 Da), lipophilicity (XLOGP between 1 and 4), hydrogen bond donors (fewer than 5), hydrogen bond acceptors (fewer than 10), topological polar surface area (less than 90 Å^2^), and molar refractivity (40–130). Compounds meeting all these criteria were selected for subsequent machine learning-based prediction.

#### 4.1.7. Chemometric Analysis of Compound Distribution in Chemical Space

We systematically evaluated the distribution in chemical space between potential transdermal compounds identified in Section 4.1.6 and the remaining compounds using PCA alongside multi-level statistical tests and visualization. After standardizing the six molecular descriptors, we performed PCA for dimensionality reduction and classified the compounds into “transdermal” and “non-transdermal” groups. For datasets with a significant MANOVA result, we used independent samples *t*-tests to assess differences in PC1 and PC2 scores and employed kernel density estimation to visualize group distribution and overlap in the reduced-dimensional space. All *t*-test results were Bonferroni corrected to control the family-wise error rate.

### 4.2. Machine Learning-Based Prediction of Potential Anti-UV Chemical Components in KU DING Tea

#### 4.2.1. Construction of the Anti-UV Drug Database

The anti-UV drug dataset was obtained from the PubChem database, a public chemical information repository launched by the National Institutes of Health (NIH) in 2004, widely used in drug discovery, virtual screening, and toxicity prediction [29]. Our search strategy included key terms closely related to UV damage, such as “anti-UV,” “antioxidant” [30], “anti-inflammatory” [31], “immunosuppressive” [32], “antihistamine” [33], “DNA repair” [34], and “skin repair” [35]. A total of 2225 drugs were selected and assigned a positive label (“1”).

For negative-label (“0”) compounds (those without anti-UV effects), a total of 2280 were selected. We selected drugs from unrelated therapeutic categories, including “antibiotics,” “antiviral,” “antidepressant,” and “anti-anxiety” agents. The number of negative-label samples was balanced to match the positive-label dataset.

#### 4.2.2. Selection of Molecular Descriptors and Learning Algorithms

Morgan fingerprints were employed as the primary molecular descriptors due to their demonstrated effectiveness in capturing topological structure–activity relationships [36]. These fingerprints generate binary bit vectors by recursively encoding atomic neighborhood information within defined molecular radii, a method originally developed for substructure and similarity searches that has proven particularly valuable for structure–activity relationship modeling [37]. The computational efficiency and robust performance [38] of Morgan fingerprints in virtual screening applications [39] made them ideally suited for this investigation [40].

Four traditional machine learning algorithms were selected for training the structure–activity relationship of drugs, namely Random Forest (RF), Support Vector Machine (SVM), Gradient Boosting (GBoost), and Logistic Regression (LR). Random Forest (RF) was selected for its ensemble Decision Tree approach, which has been demonstrated to enhance predictive generalization capabilities by aggregating votes from all trees to generate predictions. The present mechanism has been demonstrated to reduce the risk of overfitting by introducing dual randomness in both samples and features, especially effective when working with low-dimensional binary feature sets [41]. Support Vector Machine (SVM) algorithms were founded on the principle of minimizing structural risk. Incorporated for their ability to transform low-dimensional features into higher-dimensional spaces using kernel functions, it seeks an optimal hyperplane with the maximum margin to separate categories. This approach demonstrates strong performance in handling high-dimensional feature spaces and complex relationships, ensuring classification accuracy [42]. Gradient Boosting (GBoost) methods were utilized for their iterative optimization capabilities in modeling complex relationships, a particularly valuable asset when handling the asymmetric nature of molecular activity prediction tasks [43]. Logistic Regression (LR) was included as a baseline model due to its proven effectiveness in binary classification problems and its ability to mitigate confounding effects through comprehensive variable association analysis [44].

The research team deliberately excluded deep learning approaches after careful consideration of several factors. While deep learning excels in processing high-dimensional data such as images or protein sequences [45] the specific characteristics of Morgan fingerprints—particularly their high-dimensional sparsity and binary nature—made traditional machine learning methods more computationally efficient and better suited for this particular application [46]. This decision was further supported by preliminary analyses indicating that the additional computational resources required for deep learning would not yield commensurate improvements in model performance for this specific task.

#### 4.2.3. Model Implementation, Performance Evaluation, and Prediction

The machine learning workflow was implemented in Python (version 3.9) using the scikit-learn library (version 1.2.0). Molecular Morgan fingerprints with a fixed length of 1024 bits were generated as input features for all compounds in the anti-UV drug dataset established in Section 4.2.1 using the RDKit cheminformatics toolkit (version 2022.09.5), with “numpy” handling numerical computations. The final dataset comprised 4505 samples, with 2225 positive labels and 2280 negative labels. The dataset was then randomly split into training set and independent test set at an 8:2 ratio using the train_test_split function, with the random seed set to 42 to ensure reproducibility.

During the model development phase, comprehensive hyperparameter optimization was conducted using GridSearchCV from the sklearn library, implementing 5-fold cross-validation with accuracy as the optimization metric for four distinct algorithms: RF, GBoost, LR, and SVM.

The evaluation framework incorporated seven key performance metrics: Accuracy (Acc.), F1-score, Area Under the Curve (AUC), Sensitivity (Sen.), Specificity (Spe.), Precision (Pre.), and Matthews Correlation Coefficient (MCC). The corresponding calculation formulas are provided in Equations (1)–(7) [47,48,49]. Beyond the quantitative metric, model performance was further elucidated through multiple visualization techniques, including learning curves, ROC curves, calibration plots, confusion matrices, and PCA dimensionality reduction results. All quantitative metrics and model parameters were systematically archived in Excel format for comprehensive documentation.

Following the conclusion of the training process, the four models demonstrating the highest levels of performance, in conjunction with their associated StandardScaler objects, were serialized using the joblib library for the purpose of persistent storage. During the prediction stage for KU DING tea constituents, Morgan fingerprints were initially computed for the chemical compounds identified in Section 4.1.6. These fingerprints were subsequently transformed using the preserved StandardScaler, followed by the application of the predict_proba method from each model to estimate their anti-UV probabilities. A probability threshold of 0.5 was established, and constituents with anti-UV probabilities ≥ 0.5 were designated as potential anti-UV bioactive constituents. All prediction results, including compound identifiers and their corresponding probability values from each model, were exported to an Excel file for subsequent analysis.

(1) $$Accuracy=\frac{TP+TN}{TP+TN+FP+FN}$$

(2) $$F1-core=\frac{2TP}{2TP+FP+FN}$$

(3) $$AUC=\int_{0}^{1}TRP(t)dFPR(t)$$

(4) $$Sensitivity=\frac{TP+TN}{TP+FN}$$

(5) $$Precision=\frac{TP}{TP+FP}$$

(6) $$Specoficity=\frac{TP}{TN+FP}$$

(7) $$MCC=\frac{TP\times TN\times FP\times FN}{\sqrt{\left(TP+FP)(TP+FN)(TN+FP)(TN+FN\right)}}$$

where *TP* (True Positive), *TN* (True Negative), *FP* (False Positive), and *FN* (False Negative).

#### 4.2.4. System Validation of Model Outputs

To comprehensively evaluate the reliability of the four models, our study performed a classification performance evaluation on the independent test set as described in Section 4.2.3. The fitting status of the models was analyzed by plotting learning curves, and the high-probability (≥0.5) anti-UV components predicted by the models were compared with the existing scientific literature in order to confirm their practical significance.

### 4.3. Network Pharmacology Analysis

The potentially transdermal-absorbed components of KU DING tea identified in Section 4.1.5 were submitted to the BATMAN-TCM database, an online platform specializing in herb–compound–target interactions that integrates chemical, genomic, and pharmacological data to support traditional Chinese medicine research and mechanistic exploration [50]. After inputting the chemical components, we applied strict filtering criteria including a score cutoff of 0.84 (LR = 80.88), adjusted *p*-value < 0.05, and druggable score ≥ 0.99, which yielded compound–target interactions.

The GeneCards database was then queried for genes associated with UV damage, applying a relevance score threshold > 1 to select biologically significant targets. Compounds predicted in Section 4.2.3 with anti-UV probabilities > 0.5 were also submitted to BATMAN-TCM to retrieve their corresponding targets. A three-way intersection analysis was performed between the targets of KU DING tea’s transdermal-absorbed compounds, targets of predicted anti-UV compounds, and UV damage-related targets from GeneCards, yielding the final candidate anti-UV targets for KU DING tea.

These targets were subsequently analyzed using Metascape to identify KEGG signaling pathways, followed by protein–protein interaction network construction through InAct database to elucidate functional roles and regulatory relationships. The original compound–target pairs were traced back to identify which KU DING tea components acted on these critical targets, forming the basis for subsequent molecular docking studies.

### 4.4. Molecular Docking Analysis

Following established molecular docking methodologies [51], we obtained 3D structures of small-molecule ligands from PubChem and protein targets from RCSB PDB for the compound–target pairs identified in Section 4.3. Docking simulations were performed using Molecular Operating Environment (MOE) 2015 software, beginning with protein preparation where endogenous ligands were removed and structures were optimized through energy minimization and protonation state adjustment.

Small-molecule ligands were imported and prepared for docking simulations. Potential binding pockets were identified using the Site Finder tool, with all relevant amino acid residues selected for subsequent semi-flexible docking using the Dock function. Post-docking analysis included recording docking scores and examining binding modes through the Ligand Interactions module, which provided detailed information on intermolecular forces including hydrogen bonds and hydrophobic interactions, as well as binding energy calculations.

The results were systematically compared with literature-reported docking studies for validation, ensuring robust prediction of KU DING tea’s bioactive compounds and their potential mechanisms in UV protection. This comprehensive approach integrated computational biology and cheminformatics methods to elucidate the molecular basis of KU DING tea’s potential anti-UV effects.

## 5. Conclusions

This study established a more robust research framework for evaluating the anti-UV effects of topical natural medicines. The results suggest that KU DING tea is a promising natural anti-UV agent, likely mediated by key components such as 4-Methoxybenzoic acid, *P*-Coumaric Acid, and Salicylic Acid. These compounds may regulate targets like MAPK14, NFKB1, and PIK3R1, thereby modulating the AGE-RAGE signaling pathway to exert protective effects.

## Figures and Tables

**Table 1:** Identification of chemical components and transdermal penetration prediction results of KU DING tea extract.

Compound	CID	Formula	Adduct/Charge	Library Score	Prediction
Protocatechuic Aldehyde	8768	C_7_H_6_O_3_	[M + H]^+^	20	√
4-Hydroxybenzoic acid	135	C_7_H_6_O_3_	[M + H]^+^	20	√
Salicylic acid	338	C_7_H_6_O_3_	[M + H]^+^	20	√
Sesamol	68289	C_7_H_6_O_3_	[M + H]^+^	20	√
Betaine	247	C_5_H_11_NO_2_	[M + H]^+^	100	
*L*-Valine	6287	C_5_H_11_NO_2_	[M + H]^+^	100	
Stachydrine	115244	C_7_H_13_NO_2_	[M + H]^+^	34.6	
Adenine	190	C_5_H_5_N_5_	[M + H]^+^	95.1	
Nicotinamide	936	C_6_H_6_N_2_O	[M + H]^+^	97.2	
Vitamin B_6_	104817	C_8_H_11_NO_3_	[M + H]^+^	93.2	
*p*-Anisic acid	7478	C_8_H_8_O_3_	[M + H]^+^	45.6	√
Methyl paraben	7456	C_8_H_8_O_3_	[M + H]^+^	45.6	√
Isovanilline	12127	C_8_H_8_O_3_	[M + H]^+^	45.6	
Methyl salicylate	4133	C_8_H_8_O_3_	[M + H]^+^	45.6	√
3-Methoxybenzoic acid	11461	C_8_H_8_O_3_	[M + H]^+^	45.6	√
Adenosine	60961	C_10_H_13_N_5_O_4_	[M + H]^+^	100	
Gastrodin	115067	C_13_H_18_O_7_	[M + H]^+^	91.4	
Salicin	439503	C_13_H_18_O_7_	[M + H]^+^	91.4	
Ginkgolide C	9867869	C_30_H_24_O_11_	[M + H]^+^	52.4	
Nodakenin	73191	C_20_H_24_O_9_	[M + H]^+^	86.4	
Hydroxytyrosol	82755	C_8_H_10_O_3_	[M + H]^+^	81.3	
Ginkgolide B	11973122	C_20_H_24_O_10_	[M + H]^+^	10.5	
Coumarin	323	C_9_H_6_O_2_	[M + H]^+^	42	√
7-*O*-Ethylmorroniside	124585685	C_22_H_34_O_14_	[M + H]^+^	13.4	
Isochlorogenic acid A	6474310	C_25_H_4_O_12_	[M + H]^+^	54.7	
Caffeine	2519	C_8_H_10_N_4_O_2_	[M + H]^+^	84.8	
Prim-*O*-glucosylicimifugin	14034912	C_22_H_28_O_11_	[M + H]^+^	88.9	
Tetrahydrojatrorrhizine	185605	C_20_H_23_NO_4_	[M + H]^+^	62.9	√
Phellodendrine	3081405	C_20_H_24_NO_4_	[M + H]^+^	62.9	√
Cimifugin	441960	C_16_H_18_O_6_	[M + H]^+^	13.1	
Pteroside A	169727	C_21_H_30_O_8_	[M + H]^+^	53.9	
Albiflorin	24868421	C_23_H_28_O_11_	[M + H]^+^	71.8	
Paeoniflorin	442534	C_23_H_28_O_11_	[M + H]^+^	71.8	
Isoorientin	114776	C_21_H_20_O_11_	[M + H]^+^	13.5	
Luteoloside	5280637	C_21_H_20_O_11_	[M + H]^+^	13.5	
Quercitrin	5280459	C_21_H_20_O_11_	[M + H]^+^	13.5	
Parthenolide	7251185	C_15_H_20_O_3_	[M + H]^+^	66.4	
Atractylenolide III	155948	C_15_H_20_O_3_	[M + H]^+^	66.4	
Lobetyolin	14655097	C_14_H_18_O_5_	[M + H]^+^	20.9	
Secoisolariciresinol	65373	C_20_H_26_O_6_	[M + H]^+^	85.4	
Betulonic acid	122844	C_30_H_46_O_3_	[M + H]^+^	13.2	
Wilforlide A	158477	C_30_H_46_O_3_	[M + H]^+^	13.2	
Andrographolide	5318517	C_20_H_30_O_5_	[M + H]^+^	83.8	
Linolenic acid	5280934	C_18_H_30_O_2_	[M + H]^+^	99.4	
*L*-Carvone	439570	C_10_H_14_O	[M + H]^+^	52.3	
Carvacrol	10364	C_10_H_14_O	[M + H]^+^	52.3	
Vitamin C	54670067	C_6_H_8_O_6_	[M + H]^+^	47.4	
Hymecromone	5280567	C_10_H_8_O_3_	[M + H]^+^	24.5	√
7-Methoxycoumarin	10748	C_10_H_8_O_3_	[M + H]^+^	24.5	√
*p*-Coumaric acid	637542	C_9_H_8_O_3_	[M + H]^+^	81.9	√
(*+*)-Syringaresinol	443023	C_22_H_26_O_8_	[M + H]^+^	29.5	
Syringaresinol	100067	C_22_H_26_O_8_	[M + H]^+^	29.5	
Pterosin G	169739	C_14_H_18_O_4_	[M + H]^+^	15.5	√
Butyl paraben	7184	C_11_H_14_O_3_	[M + H]^+^	14.2	
Dihydrocapsaicin	107982	C_18_H_29_NO_3_	[M + H]^+^	15.5	
Dehydrodiisoeugenol	5379033	C_20_H_20_O_4_	[M + H]^+^	43.3	
Levistolide A	70698035	C_24_H_28_O_4_	[M − H]^−^	57.2	
Curcumenol	167812	C_15_H_22_O_2_	[M − H]^−^	100	
Ethyl ferulate	736681	C_12_H_14_O_4_	[M − H]^−^	79.1	√
Raspberry Ketone	21648	C_10_H_12_O_2_	[M − H]^−^	31.1	√
Curcumol	14240392	C_15_H_24_O_2_	[M − H]^−^	27.9	
Curdione	6441391	C_15_H_24_O_2_	[M − H]^−^	21.1	
Deoxycholic acid	222528	C_24_H_40_O_4_	[M − H]^−^	82.8	
*L*-Malic acid	222656	C_4_H_6_O_5_	[M − H]^−^	97.6	
Isoscopoletin	69894	C_1O_H_8_O_4_	[M − H]^−^	96.9	√
Scopoletin	5280460	C_10_H_8_O_4_	[M − H]^−^	96.9	√
Dictamnine	68085	C_12_H_9_NO_2_	[M − H]^−^	70.6	√
Quinic acid	6508	C_7_H_12_O_6_	[M − H]^−^	96.9	
Cantharidin	5944	C_10_H_12_O_4_	[M − H]^−^	25.4	
Dracorhodin	69509	C_15_H_12_O_3_	[M − H]^−^	86.4	√
Citric acid	311	C_6_H_8_O_7_	[M − H]^−^	52.2	
Aucubin	91458	C_15_H_22_O_9_	[M − H]^−^	100	
7-Ethyl-10-hydroxycamptothecin	104842	C_22_H_20_N_2_O_5_	[M − H]^−^	100	
Massoniresionl	91885243	C_20_H_24_O_8_	[M − H]^−^	100	
Borneol acetate	93009	C_12_H_20_O_2_	[M − H]^−^	30.6	

Note: “√” means compounds predicted to have potential transdermal absorption.

**Table 2:** Performance indicators of four machine learning anti-UV prediction models.

Model	Acc.	F1 Score	AUC	Sen.	Spe.	Pre.	MCC
RF	0.84	0.84	0.93	0.85	0.83	0.86	0.68
GBoost	0.84	0.84	0.91	0.86	0.82	0.86	0.68
LR	0.80	0.80	0.86	0.81	0.78	0.83	0.59
SVM	0.83	0.83	0.91	0.84	0.83	0.86	0.66

**Table 3:** Machine learning prediction results of Kudingcha anti-UV chemical components (transdermal).

Compound	RF	GBoost	LR	SVM	Average
Salicylic acid	1.00	1.00	1.00	1.00	1.00
Methyl salicylate	1.00	1.00	1.00	0.99	1.00
Raspberry Ketone	0.94	0.94	0.99	0.95	0.96
*p*-Coumaric acid	0.97	0.95	0.95	0.94	0.95
4-Hydroxybenzoic acid	0.98	0.91	0.96	0.96	0.95
Protocatechuic Aldehyde	0.89	0.97	0.99	0.92	0.94
Methyl paraben	0.89	0.95	1.00	0.91	0.94
Sesamol	0.89	0.86	0.94	0.91	0.90
*p*-Anisic acid	0.88	0.81	0.81	0.87	0.84
Ethyl ferulate	0.93	0.90	0.54	0.91	0.82
3-Methoxybenzoic acid	0.84	0.86	0.84	0.56	0.77
Dracorhodin	0.59	0.87	0.80	0.56	0.71
Phellodendrine	0.55	0.25	0.91	0.60	0.58
Scopoletin	0.47	0.32	0.51	0.56	0.46
Isoscopoletin	0.46	0.21	0.38	0.63	0.42
Hymecromone	0.40	0.31	0.48	0.45	0.41
Tetrahydrojatrorrhizine	0.56	0.04	0.50	0.34	0.36
Pterosin G	0.38	0.67	0.13	0.23	0.35
Dictamnine	0.44	0.57	0.05	0.28	0.34
7-Methoxycoumarin	0.42	0.20	0.44	0.21	0.32
Coumarin	0.19	0.16	0.46	0.09	0.22

**Table 4:** Details of molecular docking results.

Ligand	Target	PDBID	Score	E (kcal/mol)	Evidence
4-Methoxybenzoic acid	MAPK14	1A9U	−3.9845	−2.0	[14]
*P*-Coumaric Acid	NFKB1	8TQD	−3.5771	−0.9	[15]
Salicylic Acid			−3.2783	−0.8	
Methyl 4-Hydroxybenzoate	PTGS2	1PTH	−4.4289	−0.4	[16]
Methyl Salicylate			−4.4497	−2.2	
Salicylic Acid	CDK4	2W96	−3.8165	−5.0	[17]

## Data Availability

The databases used in this study are all public databases, and citations should be noted when referenced. The databases include BATMAN-TCM (“https://bionet.ncpsb.org.cn/batman-tcm/#/search (accessed on 12 March 2025)”), Pubchem (“https://pubchem.ncbi.nlm.nih.gov/ (accessed on 25 February 2025)”), RCSB PDB (“https://www.rcsb.org/ (accessed on 12 March 2025)”). Swiss target prediction(“https://www.swisstargetprediction.ch/ (accessed on 21 March 2025)”).

## References

[1] Rastogi R.P., Richa, Kumar A., Tyagi M.B., Sinha R.P. (2010). Molecular mechanisms of ultraviolet radiation-induced DNA damage and repair. J. Nucleic Acids.

[2] Kciuk M., Marciniak B., Mojzych M., Kontek R. (2020). Focus on UV-Induced DNA Damage and Repair-Disease Relevance and Protective Strategies. Int. J. Mol. Sci..

[3] Wnuk W., Michalska K., Krupa A., Pawlak K. (2022). Benzophenone-3, a chemical UV-filter in cosmetics: Is it really safe for children and pregnant women?. Postep. Dermatol. Alergol..

[4] Hohl M., Götzinger F., Jäger S., Wagmann L., Tokcan M., Tschernig T., Reichrath J., Federspiel J.M., Boor P., Meyer M.R. (2025). Assessing phototoxic drug properties of hydrochlorothiazide using human skin biopsies. Commun. Biol..

[5] Wüpper S., Lüersen K., Rimbach G. (2020). Chemical Composition, Bioactivity and Safety Aspects of Kuding Tea-From Beverage to Herbal Extract. Nutrients.

[6] Ling X., Zhou J., Jin T., Xu W., Sun X., Li W., Ding Y., Liang M., Zhu C., Zhao P. (2022). Acteoside attenuates RSV-induced lung injury by suppressing necroptosis and regulating metabolism. Front. Pharmacol..

[7] Li F., Xiao L., Lin X., Dai J., Hou J., Wang L. (2023). Deep Eutectic Solvents-Based Ultrasound-Assisted Extraction of Antioxidants from Kudingcha (llex kudingcha C.J. Tseng): Process Optimization and Comparison with Other Methods. Foods.

[8] Zhou J., Yi H., Zhao Z.X., Shang X.Y., Zhu M.J., Kuang G.J., Zhu C.C., Zhang L. (2018). Simultaneous qualitative and quantitative evaluation of Ilex kudingcha C. J. tseng by using UPLC and UHPLC-qTOF-MS/MS. J. Pharm. Biomed. Anal..

[9] Mu J., Yang F., Tan F., Zhou X., Pan Y., Long X., Zhao X. (2020). Determination of Polyphenols in Ilex kudingcha and Insect Tea (Leaves Altered by Animals) by Ultra-high-performance Liquid Chromatography-Triple Quadrupole Mass Spectrometry (UHPLC-QqQ-MS) and Comparison of Their Anti-Aging Effects. Front. Pharmacol..

[10] Liu J., Ma X., Xing J., Shi W. (2020). Anti-ultraviolet property of silk fabric dyed by flavonoids extraction from broadleaf holly leaf. J. Silk.

[11] Ma Y., Li Y., Yao Y., Huang T., Lan C., Li L. (2025). Mechanistic studies on protective effects of total flavonoids from Ilex latifolia Thunb. on UVB-radiated human keratinocyte cell line (HaCaT cells) based on network pharmacology and molecular docking technique. Photochem. Photobiol..

[12] Issa N.T., Stathias V., Schürer S., Dakshanamurthy S. (2021). Machine and deep learning approaches for cancer drug repurposing. Semin. Cancer Biol..

[13] Zhang P., Zhang D., Zhou W., Wang L., Wang B., Zhang T., Li S. (2023). Network pharmacology: Towards the artificial intelligence-based precision traditional Chinese medicine. Brief. Bioinform..

[14] Wang Z., Canagarajah B.J., Boehm J.C., Kassisà S., Cobb M.H., Young P.R., Abdel-Meguid S., Adams J.L., Goldsmith E.J. (1998). Structural basis of inhibitor selectivity in MAP kinases. Structure.

[15] Takahashi M., Chong H.B., Zhang S., Yang T.Y., Lazarov M.J., Harry S., Maynard M., Hilbert B., White R.D., Murrey H.E. (2024). DrugMap: A quantitative pan-cancer analysis of cysteine ligandability. Cell.

[16] Loll P.J., Picot D., Garavito R.M. (1995). The structural basis of aspirin activity inferred from the crystal structure of inactivated prostaglandin H2 synthase. Nat. Struct. Biol..

[17] Day P.J., Cleasby A., Tickle I.J., O’Reilly M., Coyle J.E., Holding F.P., McMenamin R.L., Yon J., Chopra R., Lengauer C. (2009). Crystal structure of human CDK4 in complex with a D-type cyclin. Proc. Natl. Acad. Sci. USA.

[18] Zhou W., Zhang H., Wang X., Kang J., Guo W., Zhou L., Liu H., Wang M., Jia R., Du X. (2022). Network pharmacology to unveil the mechanism of Moluodan in the treatment of chronic atrophic gastritis. Phytomedicine.

[19] Yee J.L., Huang C.Y., Yu Y.C., Huang S.J. (2024). Potential Mechanisms of Guizhi Fuling Wan in Treating Endometriosis: An Analysis Based on TCMSP and DisGeNET Databases. J. Ethnopharmacol..

[20] Ru J., Li P., Wang J., Zhou W., Li B., Huang C., Li P., Guo Z., Tao W., Yang Y. (2014). TCMSP: A database of systems pharmacology for drug discovery from herbal medicines. J. Cheminform..

[21] Lin Y., Chen X.J., He L., Yan X.L., Li Q.R., Zhang X., He M.H., Chang S., Tu B., Long Q.D. (2023). Systematic elucidation of the bioactive alkaloids and potential mechanism from Sophora flavescens for the treatment of eczema via network pharmacology. J. Ethnopharmacol..

[22] Du H., Zhang L., Sun H., Zheng S., Zhang H., Yuan S., Zhou J., Fang Z., Song J., Mei M. (2024). Exploring the Underlying Mechanisms of Qingxing Granules Treating H1N1 Influenza Based on Network Pharmacology and Experimental Validation. Pharmaceuticals.

[23] Nair R.S., Billa N., Morris A.P. (2025). Optimizing In Vitro Skin Permeation Studies to Obtain Meaningful Data in Topical and Transdermal Drug Delivery. AAPS PharmSciTech.

[24] Zhu F., Cai Y.Z., Sun M., Ke J., Lu D., Corke H. (2009). Comparison of major phenolic constituents and in vitro antioxidant activity of diverse Kudingcha genotypes from Ilex kudingcha, Ilex cornuta, and Ligustrum robustum. J. Agric. Food Chem..

[25] Tropsha A., Isayev O., Varnek A., Schneider G., Cherkasov A. (2024). Integrating QSAR modelling and deep learning in drug discovery: The emergence of deep QSAR. Nat. Rev. Drug Discov..

[26] Semchyshyn H. (2024). Fructose-mediated AGE-RAGE axis: Approaches for mild modulation. Front. Nutr..

[27] Wang B., Jiang T., Qi Y., Luo S., Xia Y., Lang B., Zhang B., Zheng S. (2024). AGE-RAGE Axis and Cardiovascular Diseases: Pathophysiologic Mechanisms and Prospects for Clinical Applications. Cardiovasc. Drugs Ther..

[28] Lee E.J., Kim J.Y., Oh S.H. (2016). Advanced glycation end products (AGEs) promote melanogenesis through receptor for AGEs. Sci. Rep..

[29] Kim S., Chen J., Cheng T., Gindulyte A., He J., He S., Li Q., Shoemaker B.A., Thiessen P.A., Yu B. (2021). PubChem in 2021: New data content and improved web interfaces. Nucleic Acids Res..

[30] Moschetti A., Fox C.A., McGowen S., Ryan R.O. (2022). Lutein nanodisks protect human retinal pigment epithelial cells from UV light-induced damage. Front. Nanotechnol..

[31] Nan W., Ding L., Chen H., Khan F.U., Yu L., Sui X., Shi X. (2018). Topical Use of Quercetin-Loaded Chitosan Nanoparticles Against Ultraviolet B Radiation. Front. Pharmacol..

[32] Valejo Coelho M.M., Matos T.R., Apetato M. (2016). The dark side of the light: Mechanisms of photocarcinogenesis. Clin. Dermatol..

[33] Fourzali K.M., Yosipovitch G. (2019). Management of Itch in the Elderly: A Review. Dermatol. Ther..

[34] Stege H., Roza L., Vink A.A., Grewe M., Ruzicka T., Grether-Beck S., Krutmann J. (2000). Enzyme plus light therapy to repair DNA damage in ultraviolet-B-irradiated human skin. Proc. Natl. Acad. Sci. USA.

[35] Wang J., Qiu H., Xu Y., Gao Y., Tan P., Zhao R., Liu Z., Tang Y., Zhu X., Bao C. (2022). The biological effect of recombinant humanized collagen on damaged skin induced by UV-photoaging: An in vivo study. Bioact. Mater..

[36] Zhong S., Guan X. (2023). Count-Based Morgan Fingerprint: A More Efficient and Interpretable Molecular Representation in Developing Machine Learning-Based Predictive Regression Models for Water Contaminants’ Activities and Properties. Environ. Sci. Technol..

[37] Rogers D., Hahn M. (2010). Extended-connectivity fingerprints. J. Chem. Inf. Model..

[38] Zhao W., Yu Y., Liu G., Liang Y., Xu D., Feng X., Guan R. (2024). MSI-DTI: Predicting drug-target interaction based on multi-source information and multi-head self-attention. Brief. Bioinform..

[39] Zhong C., Ai J., Yang Y., Ma F., Sun W. (2022). Small Molecular Drug Screening Based on Clinical Therapeutic Effect. Molecules.

[40] Fang Y., Pan X., Shen H.B. (2023). De novo drug design by iterative multiobjective deep reinforcement learning with graph-based molecular quality assessment. Bioinformatics.

[41] El-Behery H., Attia A.F., El-Fishawy N., Torkey H. (2022). An ensemble-based drug-target interaction prediction approach using multiple feature information with data balancing. J. Biol. Eng..

[42] Ding C., Bao T.Y., Huang H.L. (2022). Quantum-Inspired Support Vector Machine. IEEE Trans. Neural Netw. Learn. Syst..

[43] Ghazwani M., Begum M.Y. (2023). Computational intelligence modeling of hyoscine drug solubility and solvent density in supercritical processing: Gradient boosting, extra trees, and random forest models. Sci. Rep..

[44] Sperandei S. (2014). Understanding logistic regression analysis. Biochem. Med..

[45] van der Velden B.H.M., Kuijf H.J., Gilhuijs K.G.A., Viergever M.A. (2022). Explainable artificial intelligence (XAI) in deep learning-based medical image analysis. Med. Image Anal..

[46] Qi Z., Chen W., Naqvi R.A., Siddique K. (2022). Designing Deep Learning Hardware Accelerator and Efficiency Evaluation. Comput. Intell. Neurosci..

[47] Atwereboannah A.A., Wu W.P., Al-Antari M.A., Yussif S.B., Ejiyi C.J., Tenagyei E.K., Kissanga G.B., Emmanuel G.S.A., Gu Y.H., Ahene E. (2025). MEN: Leveraging explainable multimodal encoding network for precision prediction of CYP450 inhibitors. Sci. Rep..

[48] Oikonomou E.K., Williams M.C., Kotanidis C.P., Desai M.Y., Marwan M., Antonopoulos A.S., Thomas K.E., Thomas S., Akoumianakis I., Fan L.M. (2019). A novel machine learning-derived radiotranscriptomic signature of perivascular fat improves cardiac risk prediction using coronary CT angiography. Eur. Heart J..

[49] Chicco D., Jurman G. (2020). The advantages of the Matthews correlation coefficient (MCC) over F1 score and accuracy in binary classification evaluation. BMC Genom..

[50] Kong X., Liu C., Zhang Z., Cheng M., Mei Z., Li X., Liu P., Diao L., Ma Y., Jiang P. (2024). BATMAN-TCM 2.0: An enhanced integrative database for known and predicted interactions between traditional Chinese medicine ingredients and target proteins. Nucleic Acids Res..

[51] Shin W.R., Um H.J., Kim Y.C., Kim S.C., Cho B.K., Ahn J.Y., Min J., Kim Y.H. (2021). Biochemical characterization and molecular docking analysis of novel esterases from *Sphingobium chungbukense* DJ77. Int. J. Biol. Macromol..

